# Supplementary figures and images for: Islands of genomic stability in the face of genetically unstable metastatic cancer
Source: PLoS One. 2024 Dec 19;19(12):e0298490. doi: 10.1371/journal.pone.0298490 (PMC11658618; doi:10.1371/journal.pone.0298490)

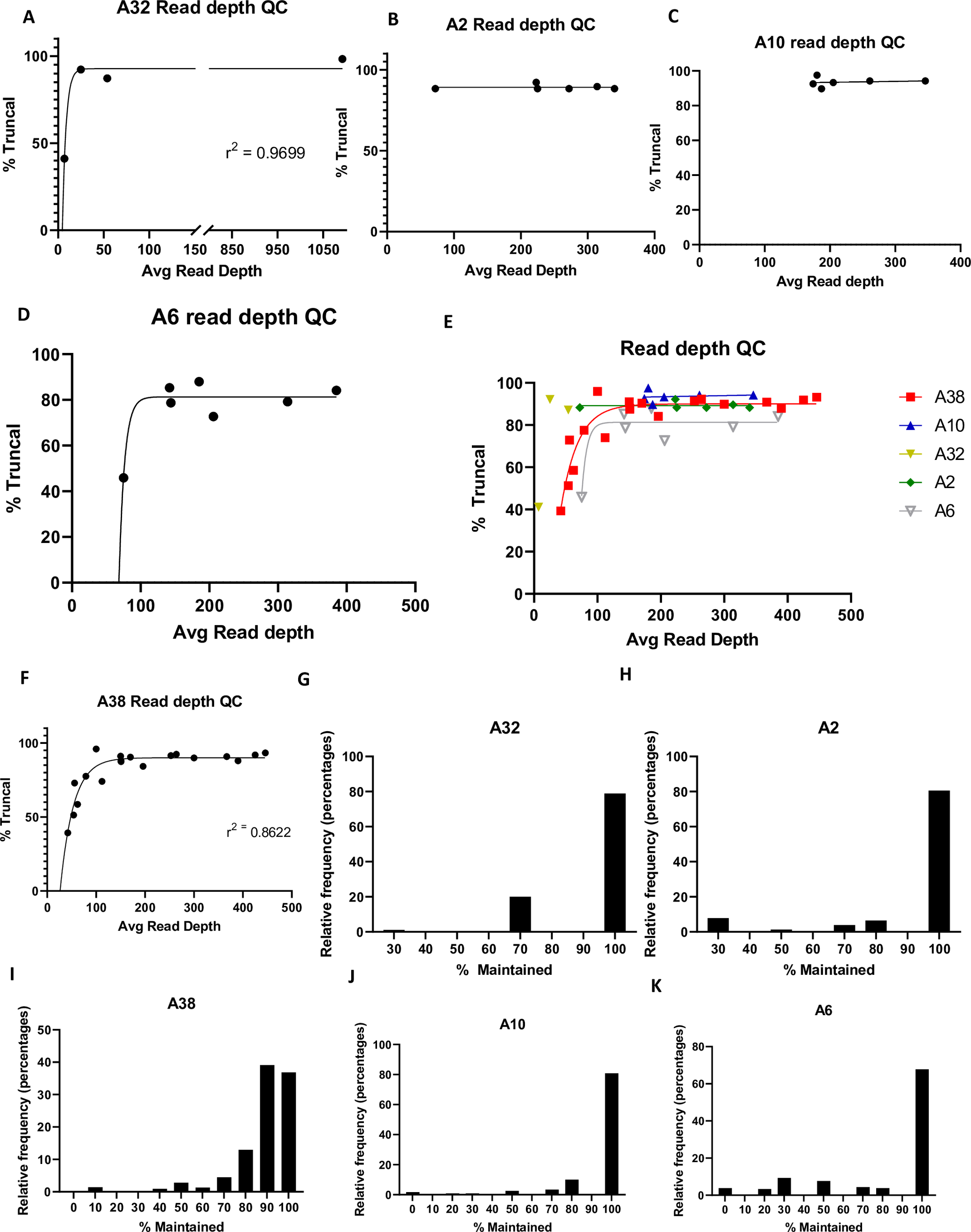

Supplement: S1 Fig — Histograms showing maintenance of PAMs for each case are also shown. (TIF) [file pone.0298490.s001.tif]

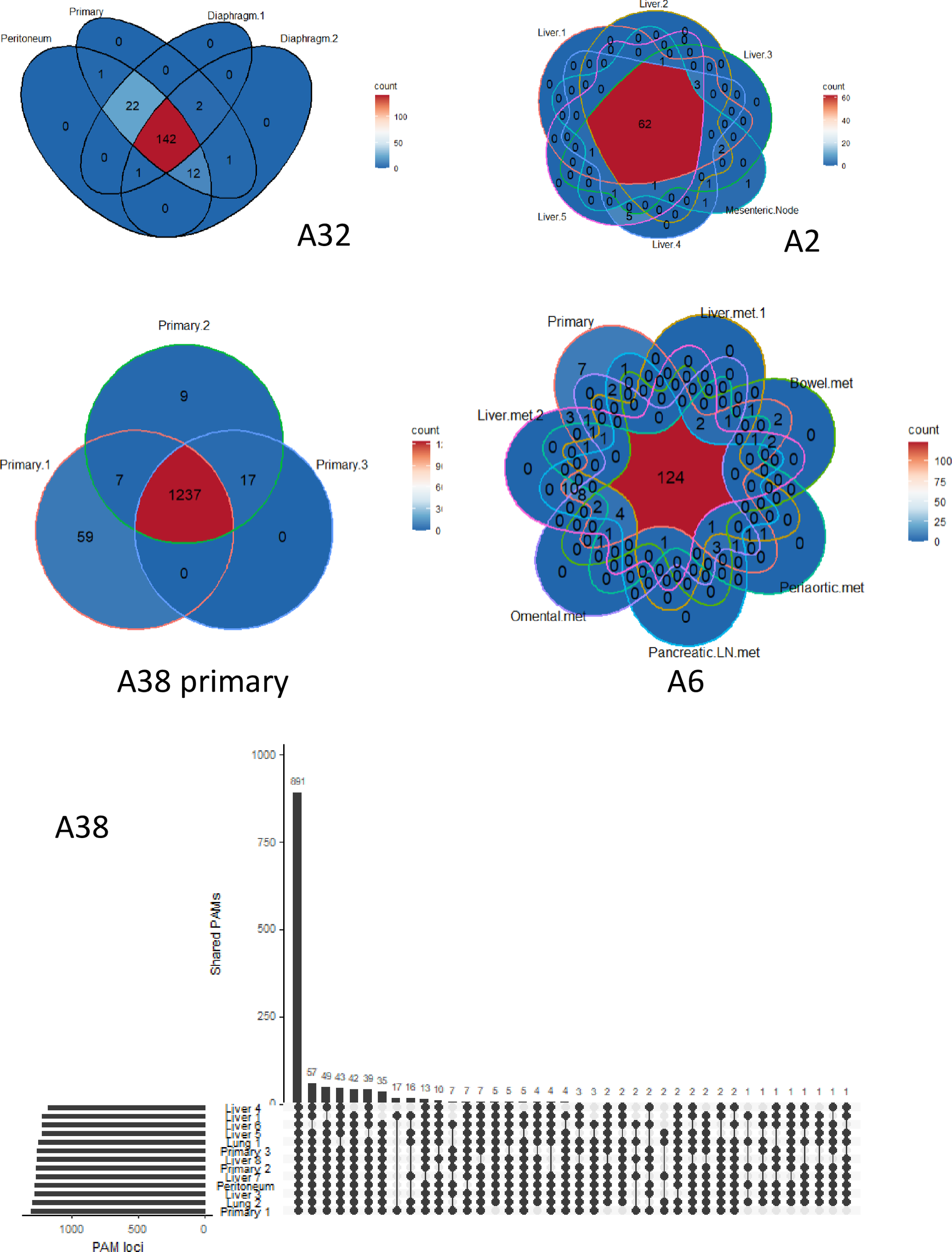

Supplement: S2 Fig — Venn diagrams were created with ggvenn from the ggplot2 R package, and the A38 subsets were plotted with UpSetR. (TIF) [file pone.0298490.s002.tif]

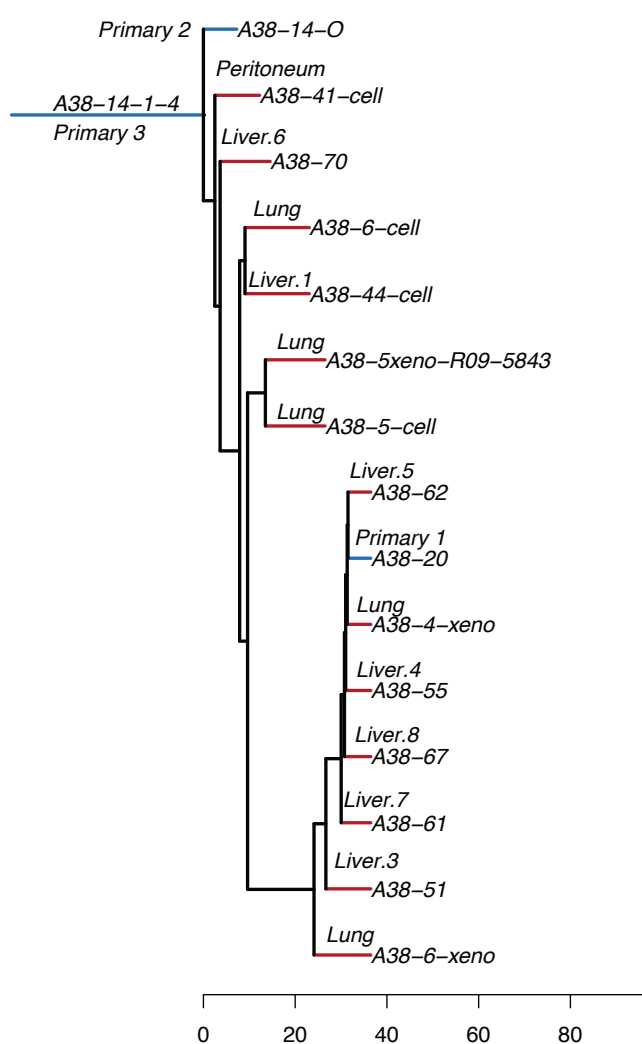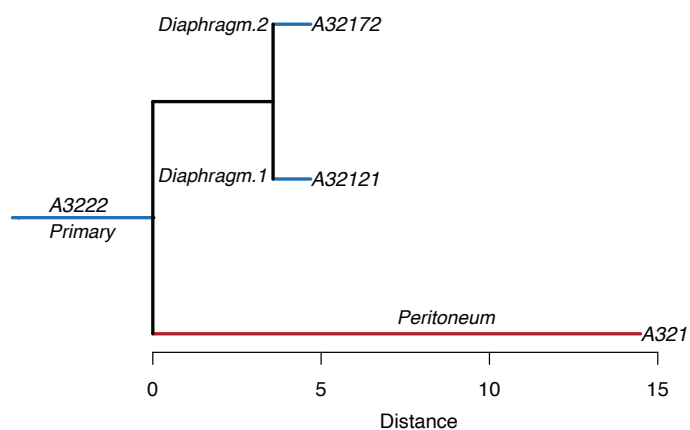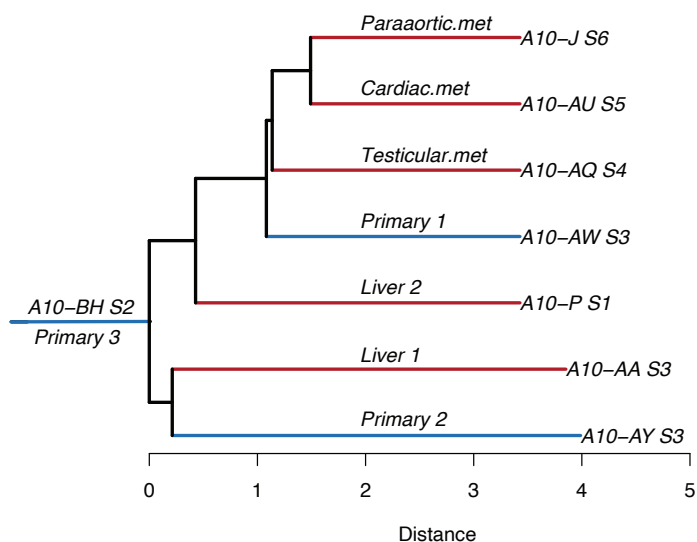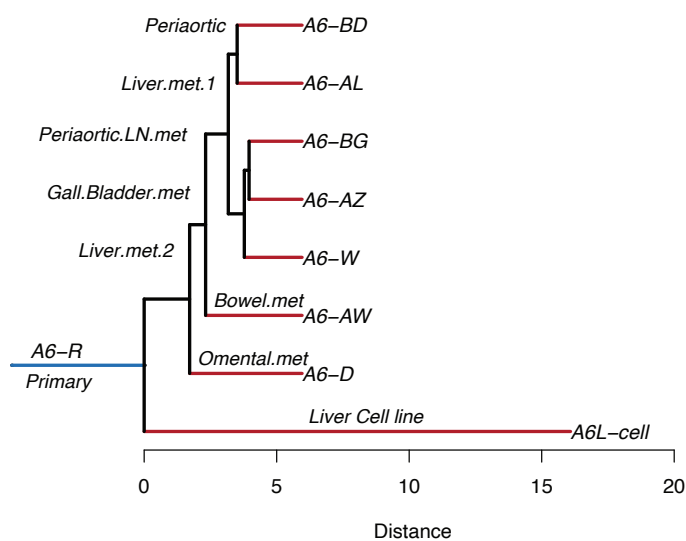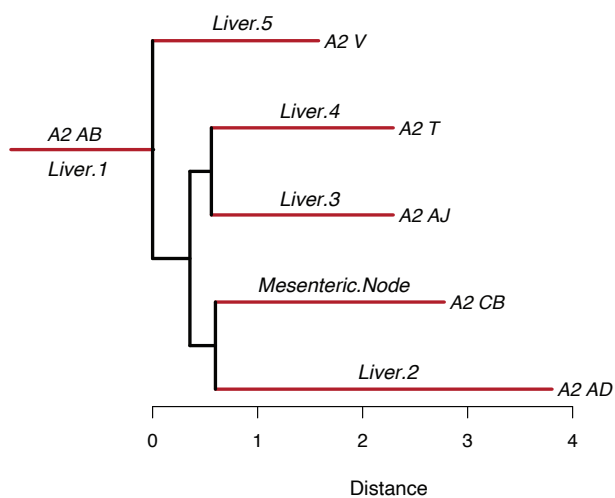

Metastatic

Primary

Supplement: S3 Fig — (PDF) [file pone.0298490.s003.pdf]

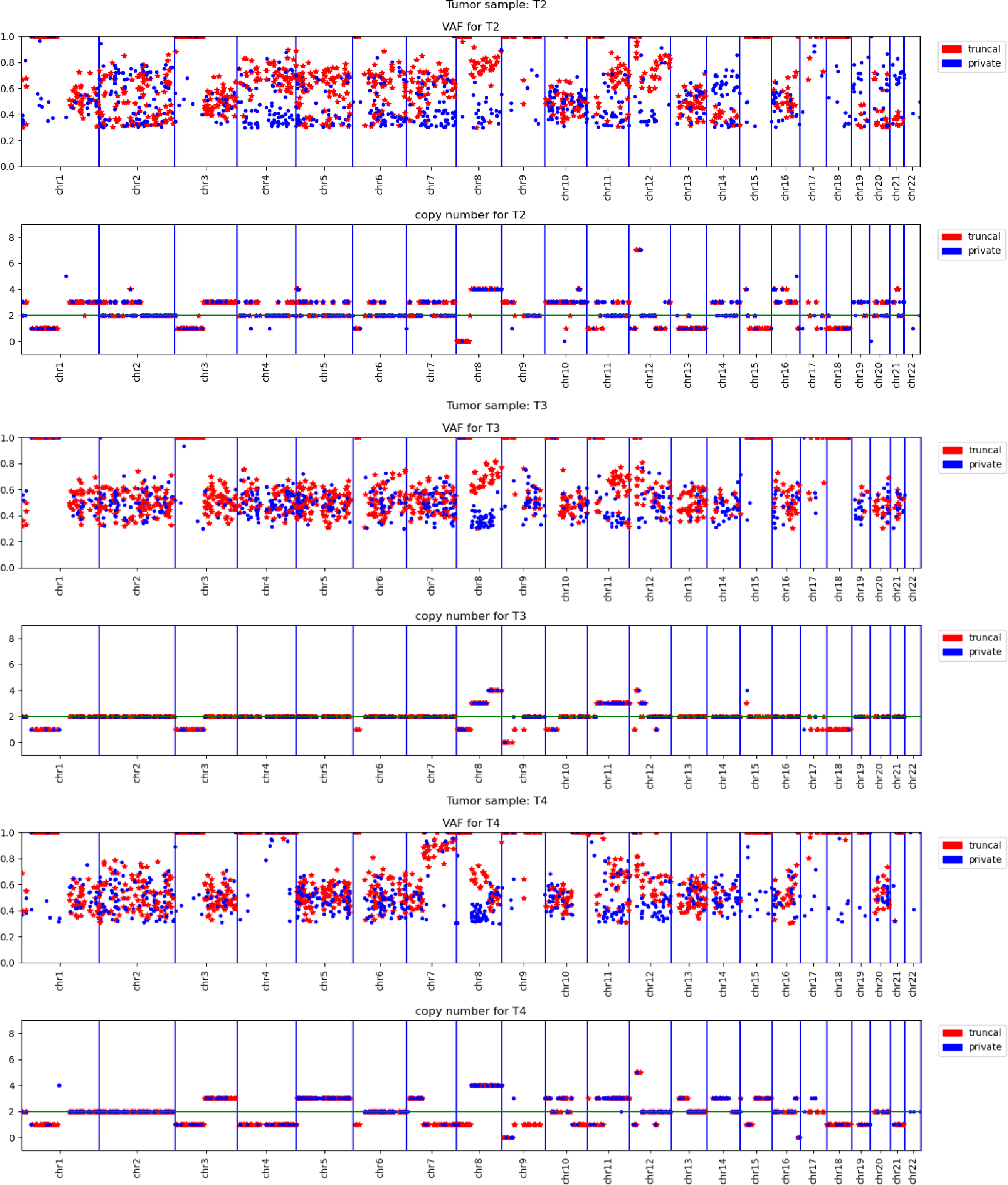

Supplement: S4 Fig — (TIF) [file pone.0298490.s004.tif]

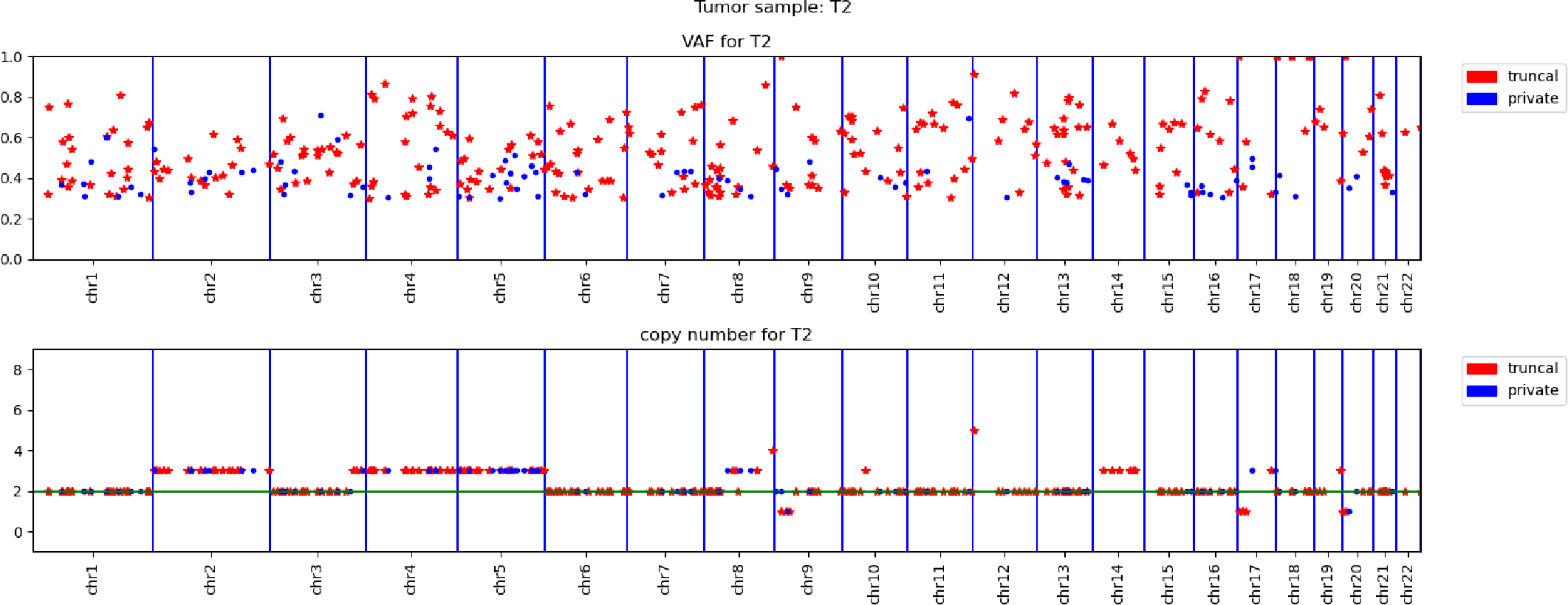

Supplement: S5 Fig — (TIF) [file pone.0298490.s005.tif]

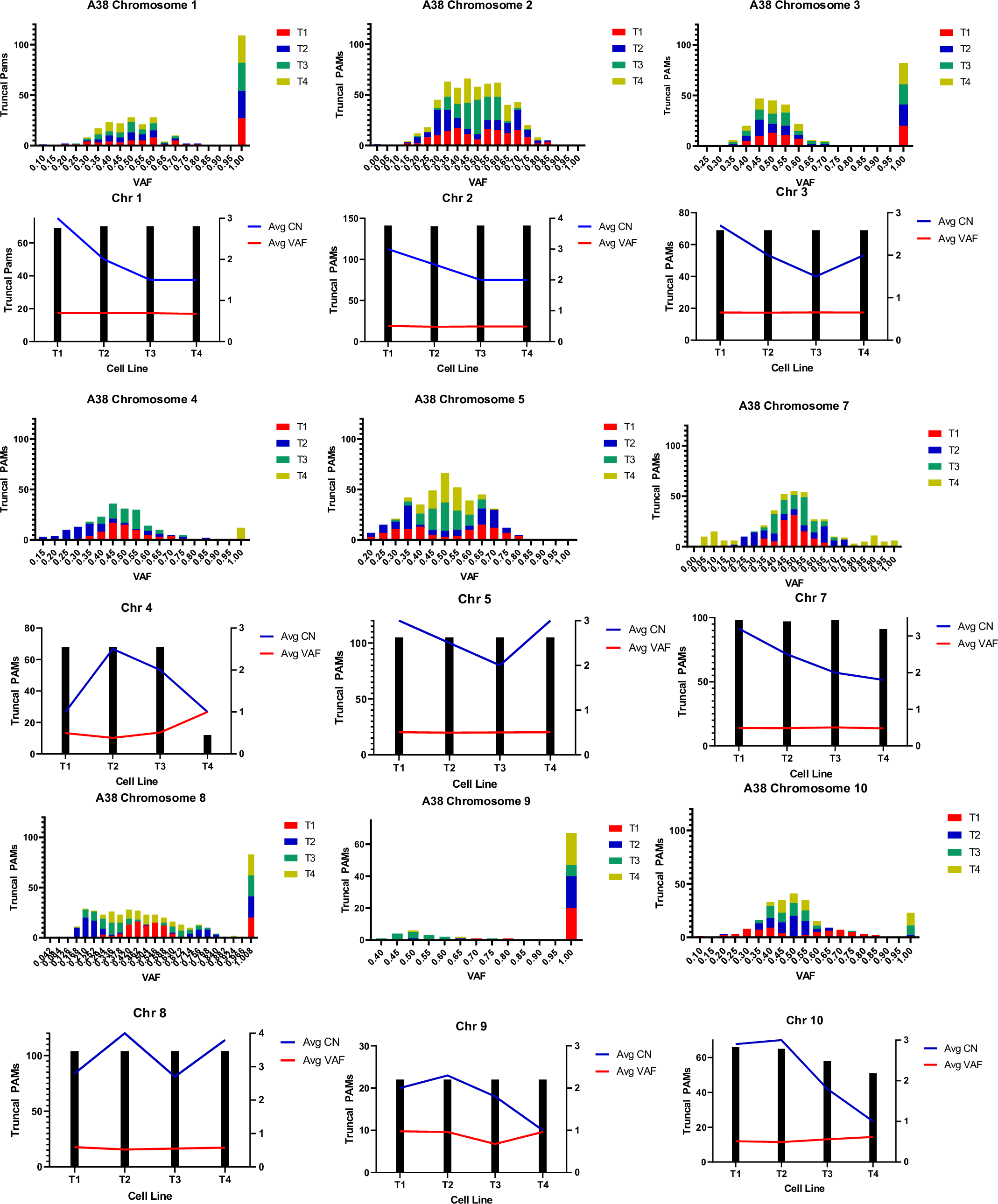

Supplement: S6 Fig — (TIF) [file pone.0298490.s006.tif]

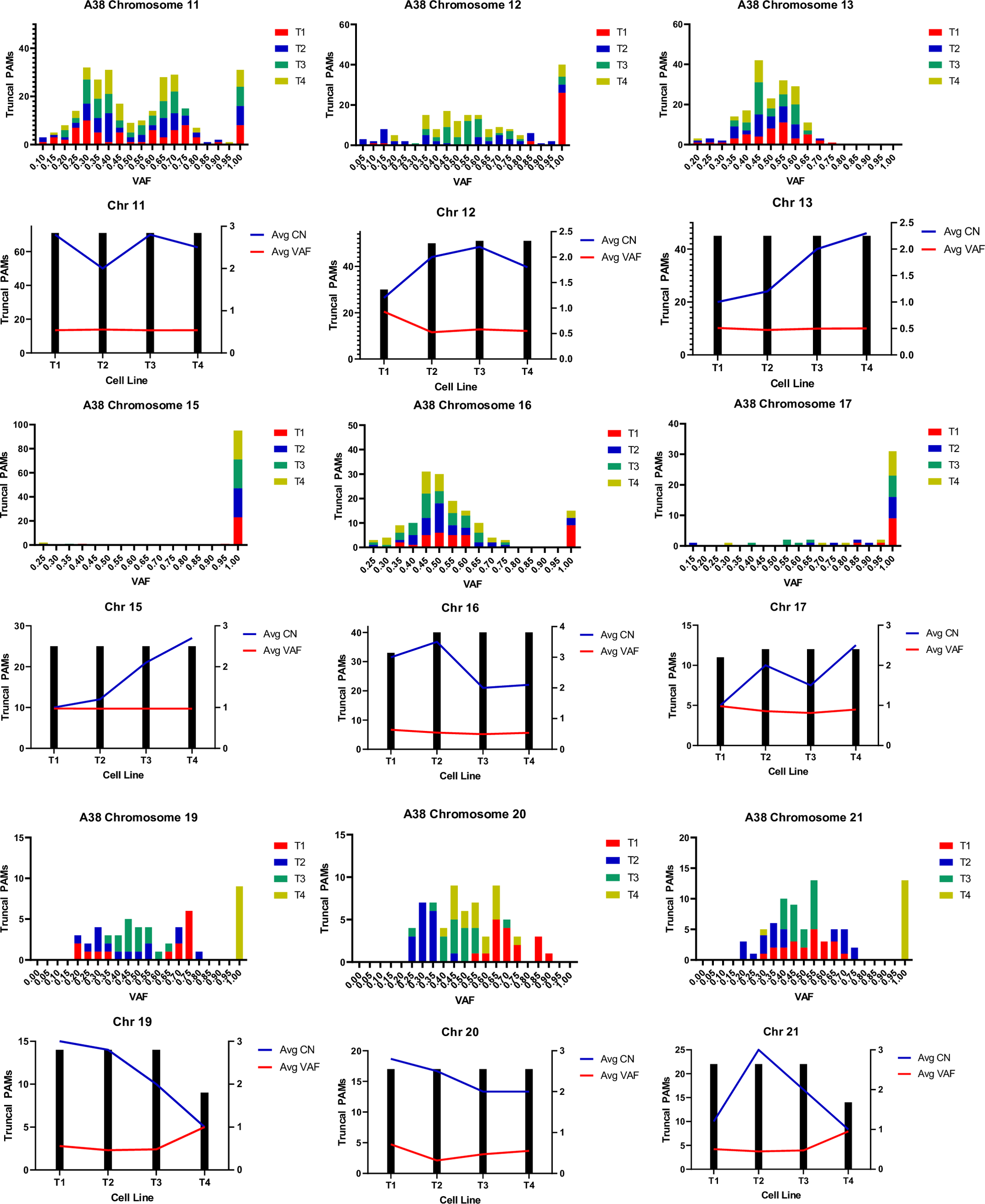

Supplement: S7 Fig — (TIF) [file pone.0298490.s007.tif]

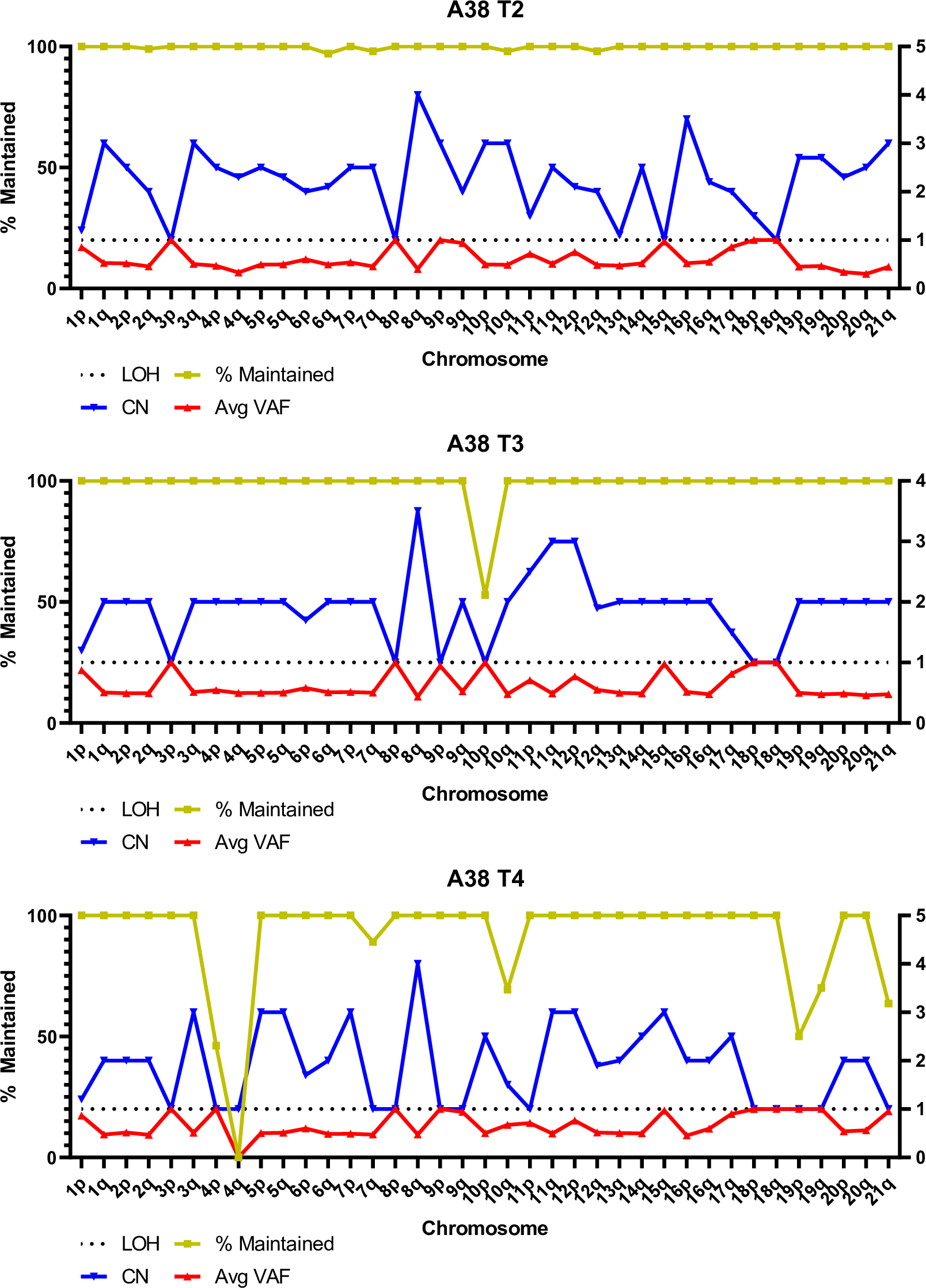

Supplement: S8 Fig — (TIF) [file pone.0298490.s008.tif]

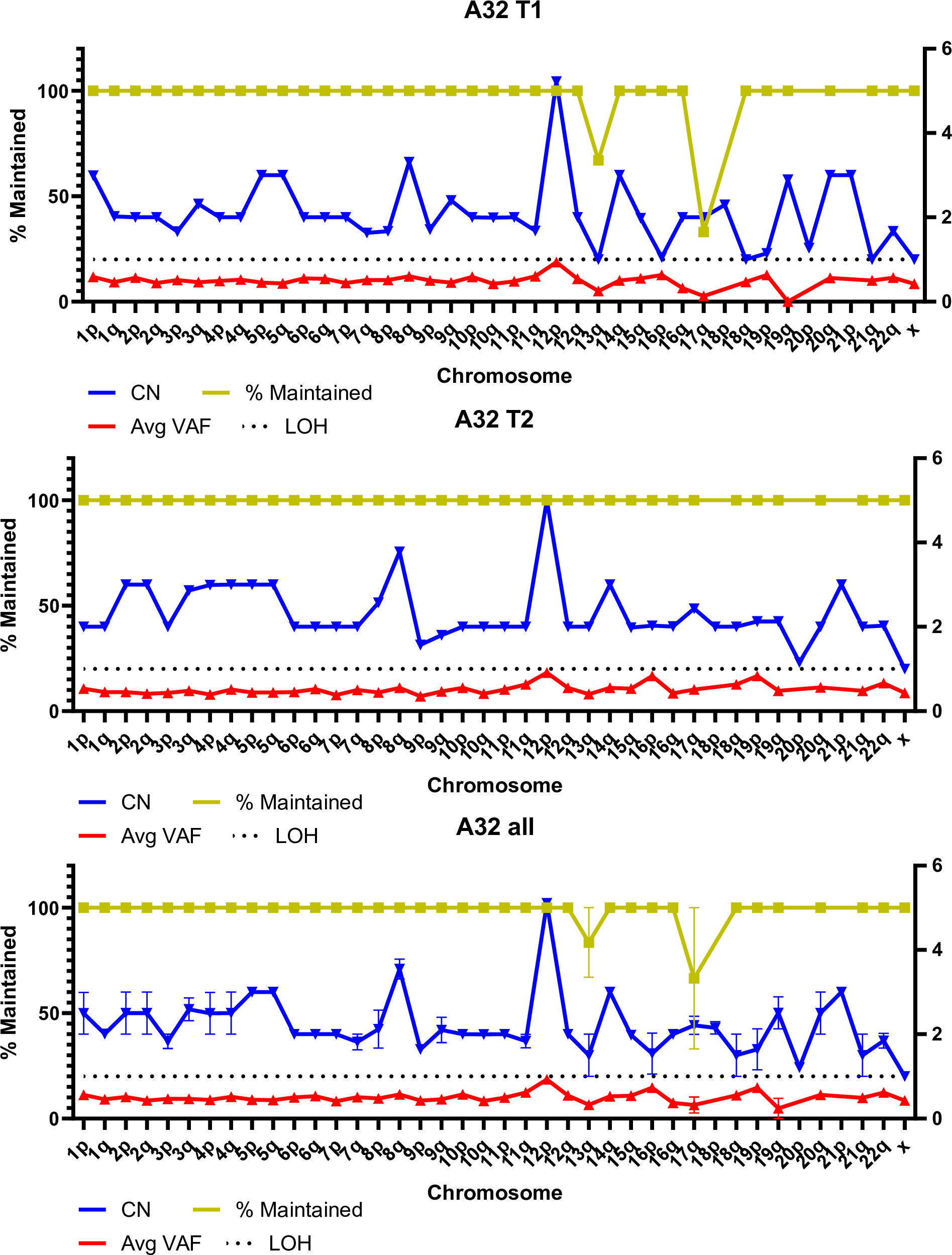

Supplement: S9 Fig — (TIF) [file pone.0298490.s009.tif]

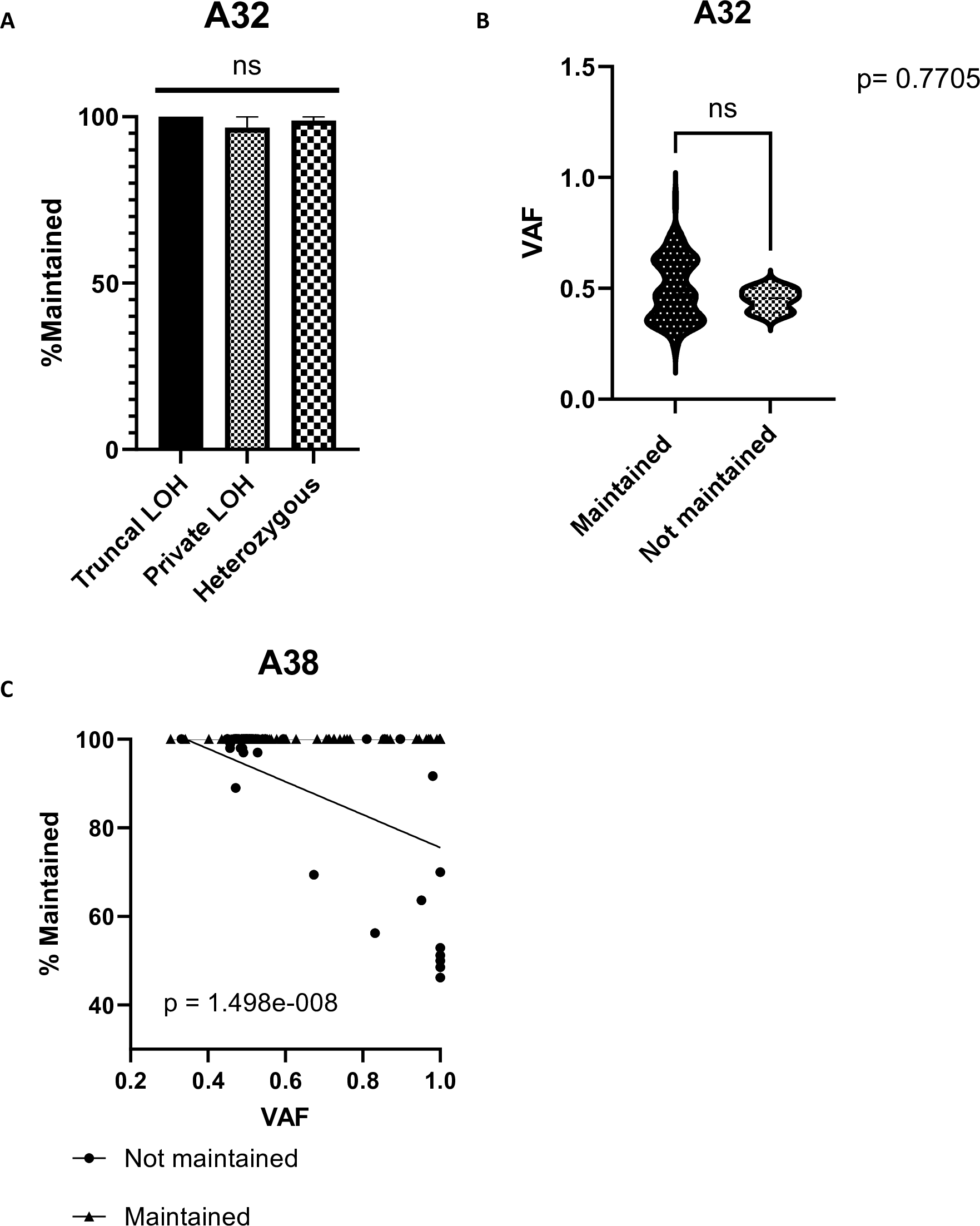

Supplement: S10 Fig — A) No significant difference was found between the two cell lines for LOH and PAM maintenance (Kruskal-Wallis, p = 0.3307) or for B) VAF between truncal PAMs maintained in both lines or lost in some (Mann-Whitney rank sum, p = 0.7705). n = 165 for maintained PAMs, n = 3 for PAMs lost in one cell line but not the other. C) Plot of truncal PAM VAF comparing PAMs maintained in all four cell lines (blue) and truncal PAMs lost in at least 1 cell line (red). A wide VAF distribution in present in PAMs present in all. For PAMs lost in some, VAFs cluster at 0.5 in samples where they are maintained, 1 when they are lost at a rate of 50%. (TIF) [file pone.0298490.s010.tif]
